# Evaluating Impact of Thermo-Oxidative and Ultraviolet Aging on Performance of Hot In-Place Recycled Asphalt Mixtures

**DOI:** 10.3390/ma17235813

**Published:** 2024-11-27

**Authors:** Yao Guan, Yao Zhang, Tianyi Sang, Yifeng Ding, Zichao Yan, Aihong Kang

**Affiliations:** 1 College of Civil Science and Engineering, Yangzhou University, Yangzhou 225127, China; yaogwhx@163.com (Y.G.); stytt7@163.com (T.S.); goethe0411@126.com (Y.D.); 18156736818@163.com (Z.Y.); 003129@yzu.edu.cn (A.K.); 2 Research Center for Basalt Fiber Composite Construction Materials, Yangzhou 225127, China

**Keywords:** asphalt pavement, hot in-place recycling technology, ultraviolet aging, pavement performance, Verhulst model, performance prediction

## Abstract

Hot in-place recycling (HIR) is a sustainable pavement rehabilitation method. However, it is susceptible to aging processes that can compromise its mechanical properties and long-term performance. This study investigates the effects of thermo-oxidative (TO) and ultraviolet (UV) aging on HIR mixtures. Basic performance tests were conducted on the aggregate gradation, moisture content, and asphalt content of the reclaimed asphalt pavement (RAP) to assess the aging level. Simulations of long-term and short-term oxidative aging of the HIR mixture, along with 12 months of UV irradiation, were performed to evaluate its high-temperature stability, low-temperature crack resistance, and water stability. The Verhulst model was employed to establish a predictive equation for performance attenuation under UV aging. To quantify the photoaging effect, indicators for UV aging degree were proposed to characterize the road performance of the HIR mixture, including the aging rate and the aging residual index. Results indicate that the improvement in high-temperature performance after aging is limited, but cracking resistance decreases substantially. Notably, the flexural tensile strain was reduced by 129.25 με for 10 years of TO aging compared to 12 months of UV exposure, underscoring the importance of considering environmental factors in performance predictions. This study emphasizes the need for enhanced aging mitigation strategies to improve the sustainability and reliability of HIR mixtures in practical applications.

## 1. Introduction

HIR pavement technology has become one of the most widely used methods in road maintenance and repair projects due to its advantages in energy efficiency, environmental sustainability, and cost reduction [1,2]. However, reclaimed asphalt pavement (RAP) deteriorates significantly during long-term service [3]. While the viscoelastic properties of asphalt can be partially restored with the addition of rejuvenators [4], recycled asphalt mixtures degrade more rapidly than new asphalt mixtures when exposed to high-temperature construction, traffic loads, and environmental factors [5,6]. These factors lead to an increased frequency of maintenance, which hampers the broader adoption and application of HIR pavement technology. Most current research focuses on the effects of aging on new asphalt mixtures and the performance enhancement of recycled asphalt mixtures. However, the impact of aging during the continued service life of recycled asphalt mixtures is seldom addressed, and the underlying aging mechanisms still need to be clarified.

During the service life of asphalt mixtures, the light fractions of asphalt undergo volatilization and cracking, leading to increased stiffness and reduced flexibility [7]. To address this, admixtures are often added during RAP regeneration to restore or improve the crack resistance of the asphalt mixture. Rejuvenators are used to replenish the light components in RAP, while fibers are incorporated to enhance the network structure of the asphalt binder [8,9].

Currently, the most commonly used recycling methods in engineering are HIR and hot mix plant recycling [10]. With advancements in hot mix plant recycling technology, the proportion of RAP in these applications is gradually increasing. However, compared to HIR, which allows for RAP blending ratios of over 70%, the economic benefits of hot mix plant recycling are limited when the RAP content is less than 50%. Although HIR offers advantages such as high energy efficiency and short construction periods [9], the performance of recycled asphalt mixtures after HIR is significantly affected by aging, primarily due to the high RAP content and secondary aging in thermal regeneration construction.

Many researchers have studied the performance effects of aging on asphalt mixtures and simulated TO and UV aging in natural engineering environments using indoor artificial accelerated aging tests [11,12]. The carbon (C) and sulfur (S) elements in asphalt undergo oxidation reactions with oxygen, and elevated temperatures accelerate these reactions. As a result, TO aging is commonly used to investigate the effects of aging on the thermal stability of styrene-butadiene-styrene (SBS)-modified asphalt [13]. Pei Z conducted TO and UV aging tests on asphalt using a pressurized aging vessel and custom UV aging equipment, revealing significant differences in the microstructure of aged asphalt [14,15]. Additionally, UV aging is a critical factor contributing to the deterioration of asphalt pavements. UV radiation severely damages the network structure formed in SBS-modified asphalt, exacerbating the degradation of SBS and causing significant changes in rheological properties following UV aging [16]. Chen Z et al. found that as UV aging progresses, asphalt films exhibit vertical gradients in molecular size and oxidation product content [17]. Some researchers have examined the combined effects of TO and UV aging on asphalt mixtures, observing that UV aging weakens asphalt cohesion and interfacial adhesion [18]. Xu Y et al. investigated the aging mechanisms of asphalt under both TO and UV conditions, concluding that composite aging leads to more severe stratification compared to single aging conditions [19].

The development of aging models is crucial for understanding the effects of aging on asphalt mixture performance and for minimizing resource waste associated with repetitive and time-consuming performance tests [20]. Nonlinear modeling has proven effective in accurately characterizing the aging rate and degree of asphalt binders [21]. Guan M et al. conducted multifactor coupling aging tests on asphalt binders using an accelerated environmental aging oven, incorporating UV radiation, temperature, and moisture. The findings indicate that the Glover-Rowe parameter serves as a reliable, comprehensive index for evaluating the rheological properties of asphalt based on correlation analysis [22]. Aging in asphalt tends to follow a gradient from the surface layer to the deeper layers of the pavement, and the Verhulst model has been shown to effectively explain the aging behavior at different nanoscale depths [23].

In this study, TO and UV aging of hot in-place recycled mixtures were simulated to reflect the actual environmental conditions of the in-serviced pavement. The high-temperature stability, low-temperature crack resistance, and water stability of mixtures after secondary aging were then evaluated. The Verhulst model was used to establish a predictive model for performance attenuation under UV aging. Additionally, UV aging indicators were proposed, including the aging rate and aging residual index, to quantify the photoaging effects on the road performance of HIR asphalt mixtures.

## 2. Raw Materials and Experimental Design

### 2.1. Overview of the Original Pavement Project

The RAP used was sourced from the HIR project on the Baoying section of the G233 national and provincial trunk road, which has a pavement design traffic load rating for heavy traffic. The project was fully completed and opened to traffic in September 2014, and the RAP had been in service for nine years at the time of sampling.

(1) Introduction of pavement distress

**Figure 1:**
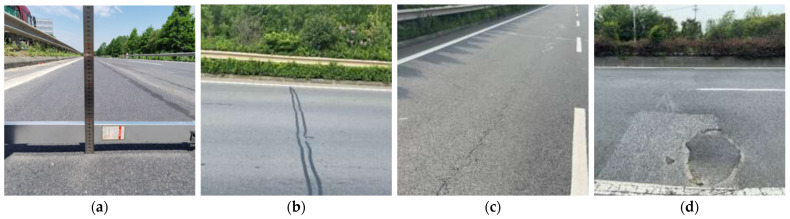
Distress types on the original pavement: (**a**) Rutting, (**b**) Transverse cracks, (**c**) Longitudinal cracks, (**d**) Pavement patching.

Figure 1 illustrates the significant rutting distress observed in the original pavement section. The primary distress type identified was transverse cracking, which accounted for 49.67% of the total affected area and exhibited a relatively dense distribution. Longitudinal cracking, comprising 15.97% of the affected area, was found across multiple sections. Field investigations indicated that substandard repair methods for localized pavement issues led to secondary problems, such as potholes and slurry formation under traffic loads. These defects not only degrade overall driving quality but also pose risks to pavement safety.

(2) Asphalt content and gradation of the original pavement

The RAP was obtained from the original pavement by heated milling, which does not broadly break the coarse aggregate particles, and the original gradation is not overly refined. Therefore, the RAP’s gradation is within the limits of the specification. Trichloroethylene was used as a solvent to remove the asphalt from the RAP. The gradation was determined using the water sieve method, as shown in Table 1. Following the “Technical Specification for Highway Asphalt Pavement Recycling”, moisture and asphalt content tests were conducted on the RAP sample. The moisture content of the coarse and fine aggregates in the RAP material was approximately 2.31% and 4.01%, respectively. The asphalt content of the reclaimed pavement was around 5.45%.

### 2.2. Raw Materials and Fundamental Properties

The raw materials primarily consist of reclaimed asphalt and aggregates from the RAP, virgin SBS-modified asphalt, basalt aggregate, and a rejuvenator sourced from Jiangsu Subote Company (Ninjing, China).

(1) Virgin and reclaimed asphalt

**Figure 2:**
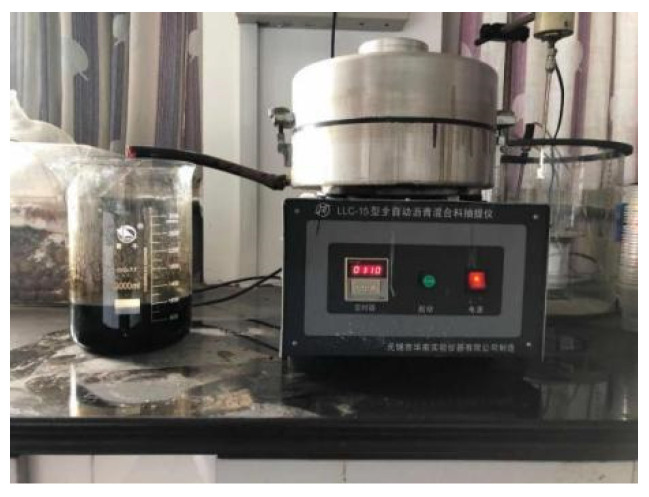
Centrifugal extraction test chart.

**Figure 3:**
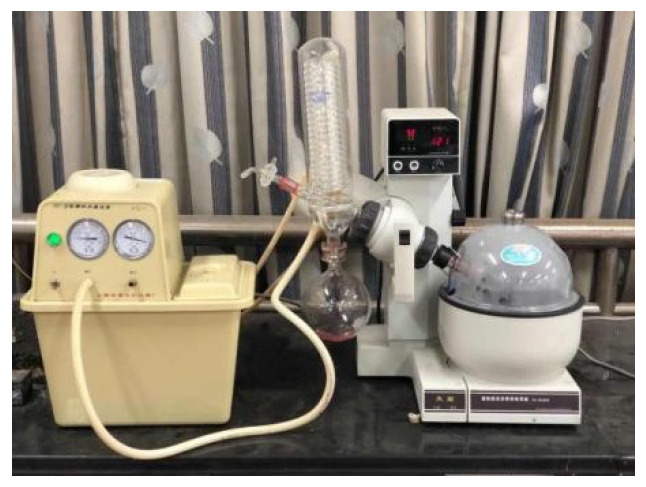
Rotational evaporation test diagram.

The reclaimed asphalt was recycled using the distillation separation method, and the technical properties of both the virgin and reclaimed asphalt were tested. The asphalt extraction instruments are shown in Figure 2 and Figure 3, and the results are presented in Table 2. The new asphalt selected SBS-modified asphalt, with a softening point, 25 °C penetration, and 5 °C ductility of 77.1 °C, 50.1 (0.1 mm), and 29.4 cm, respectively. The performance of reclaimed asphalt is inferior to that of virgin asphalt, with a 5 °C ductility of only 8.6 cm.

According to Table 2, the aging grade of RAP is determined based on needle penetration and the viscosity index. As shown in Table 3, the RAP used in this study belongs to aging grade II, and its performance can be partially improved by adding the rejuvenator.

(2) Rejuvenator

The rejuvenator (RA-102) was selected from Jiangsu Subote Company. It features a flash point of 252 °C and a 90 °C viscosity of 4000 cP. The viscosity ratio before and after the rolling thin film oven test (RTFOT) is 1.64, and the change in mass before and after the RTFOT is 1.14%. The technical properties and specification requirements are presented in Table 4 and satisfy the standard request.

(3) Mineral properties

Both the coarse and fine aggregates are basalt. The coarse aggregates are clean, rough, and free of impurities, with their physical and mechanical properties meeting the requirements and relative density, as shown in Table 5. The particle size distribution of the fine aggregate must adhere to the design specifications, and it should be clean and free from impurities such as soil, clay, and organic matter. The relative density of fine aggregates is provided in Table 6.

Limestone mineral powder was used as filler, and the performance indexes are shown in Table 7. The water content is 0.38%, the relative density is 2.769 kg/m^3^, and the particle size range satisfies the standard request.

### 2.3. Mix Design of AC-13 Hot In-Place Recycling Mixture

The original pavement gradation used a nominal maximum particle size of 13.2 mm (AC-13), which was selected for the gradation design. Based on actual engineering conditions and the Marshall test, the optimum asphalt content for the HIR mixture was determined.

(1) Determination of rejuvenator content

**Figure 4:**
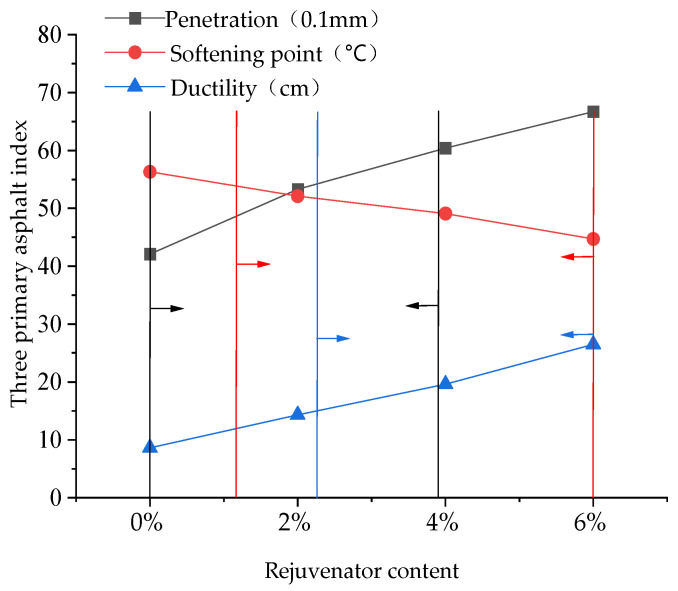
Effect of rejuvenator dosage on asphalt properties.

The rejuvenator was added to the reclaimed asphalt at 2%, 4%, and 6% by weight of the reclaimed asphalt, and tests on penetration, softening point, and ductility were performed. The test results are presented in Figure 4.

According to the specification requirements, due to the process characteristics of hot recycling, the virgin asphalt grade of the pavement is typically reduced by one level to determine the target grade for reclaimed asphalt. This corresponds to a penetration index of 40–60 (0.1 mm), which is associated with a rejuvenator content of 0–3.9%; a softening point of no less than 49 °C, corresponding to a rejuvenator dosage of 1.2–6%; and ductility of no less than 15 cm, corresponding to a rejuvenator dosage of 2.3–6%. To meet these performance requirements, a rejuvenator content range of 2.3% to 3.9% was selected. Considering the high-temperature performance and operability of rejuvenated asphalt, a rejuvenator content of 3% was ultimately selected for subsequent testing in this study.

(2) Rejuvenated asphalt mixture mix design

**Figure 5:**
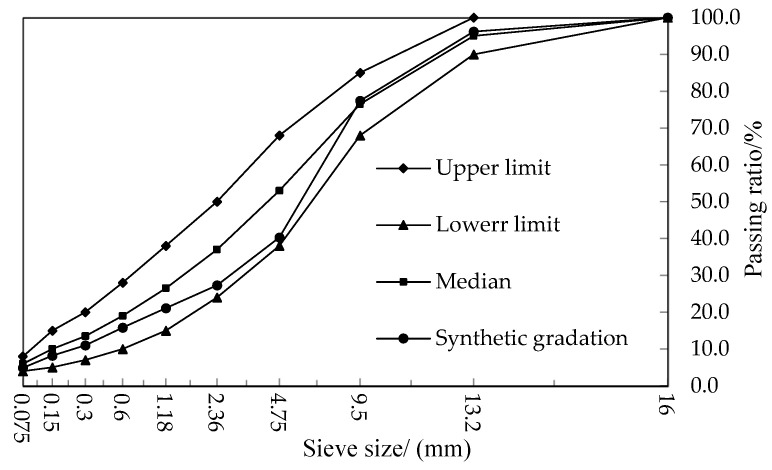
Mixture gradation curve.

Based on the actual engineering conditions, the original pavement in the section exhibited significant rutting. Therefore, the gradation at the lower limit of the specification was chosen as the final composite gradation, and the gradation diagram is shown in Figure 5. The Marshall test determined that the optimum asphalt content for the HIR mixture was 4.67%.

### 2.4. Design of Thermo-Oxidative and Ultraviolet Aging Tests

Pavement aging typically involves two mechanisms: TO aging and UV aging. TO aging occurs when asphalt pavement is exposed to high temperatures and oxygen over extended periods. This leads to the volatilization of lightweight components and changes in the molecular structure due to the combined effects of heat and oxygen. These changes increase the viscosity and elastic modulus of the asphalt while reducing its ductility, ultimately making the mixture harder and more brittle [24]. UV aging, on the other hand, results from prolonged exposure to sunlight. Intense UV irradiation causes the asphalt to become brittle and reduces its viscosity [18,25], which eventually contributes to pavement distress.

**Figure 6:**
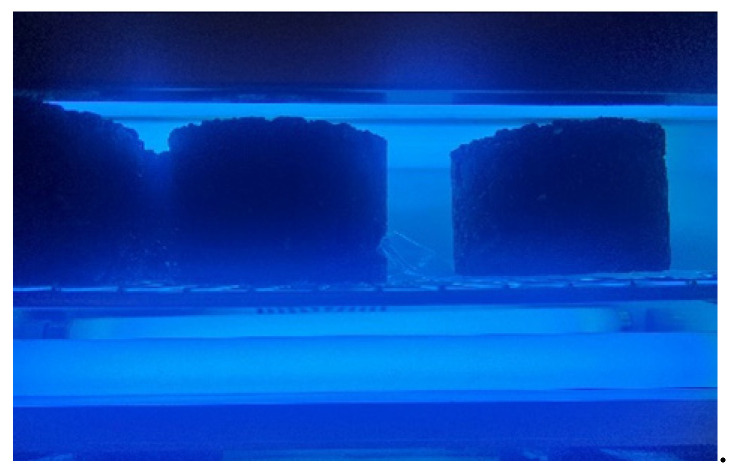
Simulation method of UV aging.

**Figure 7:**
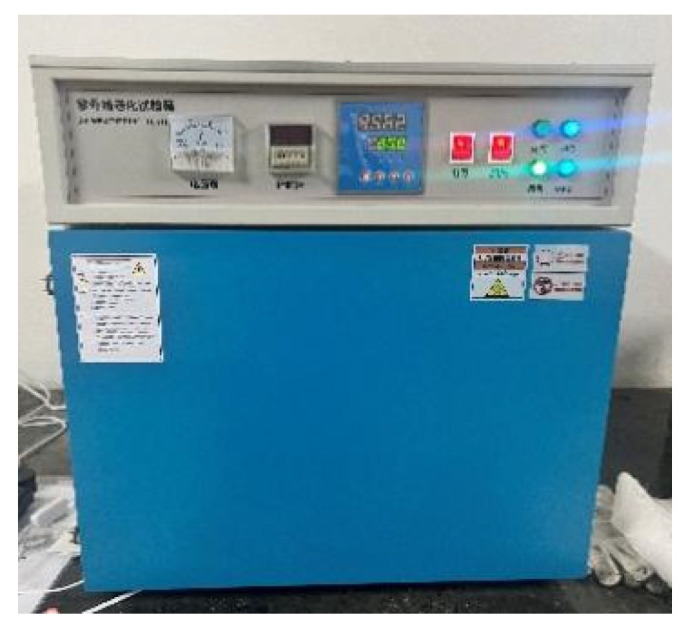
UV aging chamber.

The power of the UV lamp primarily determines the intensity of UV radiation, while the duration of exposure directly impacts the accuracy of the test results [26]. To simulate long-term exposure to sunlight, the UV radiation produced by the equipment should be at least ten times stronger than that of the outdoor environment [27]. In this study, an ultraviolet aging chamber was used to irradiate the samples, providing a more accurate simulation of UV aging under natural environmental conditions. The maximum wavelength is 365 nm, the minimum is 313 nm, and the power consists of one 300 W lamp, two 80 W lamps, and two 40 W lamps. The UV aging simulations are shown in Figure 6 and Figure 7.

The laboratory aging time to simulate actual radiation exposure was calculated using the equivalent conversion method for radiation exposure [28], as detailed in Equation (1).

(1) $$t=\frac{F\times b}{3600\times Q}$$

where *F* is the total annual sunlight radiation (J/m^2)^; *b* is the proportion of UV rays in sunlight, which is taken as 7%; *Q* is the radiation intensity of the UV lamp (W/m^2)^; and *t* is the adequate time for the conversion of natural radiation to indoor radiation, the unit is h.

Previous studies have shown that the effect of UV aging on asphalt pavement is most pronounced during the early service period [16]. In this study, the HIR mixture specimens were aged for 27, 54, 81, 108, 135, and 162 h to simulate UV exposure corresponding to 2, 4, 6, 8, 10, and 12 months of service in the city of Yangzhou, Jiangsu Province, China, respectively, as detailed in Table 8.

Hence, this study simulates the actual aging of rejuvenated pavement by varying both aging methods and duration. Among them, short-term aging at 135 °C and 155 °C was used to simulate multiple high-temperature heating phases of HIR mixtures during the construction process, and long-term aging for 120 h and 240 h simulated natural aging for 5 years and 10 years, respectively [29]. A control group without aging and another with eight different aging schemes were established, as shown in Table 9.

## 3. Performance Tests—Analysis and Prediction

To evaluate the changes in the HIR mixture following TO and UV aging, this study systematically investigated their high-temperature, low-temperature, and moisture damage properties. The evaluation was conducted through a series of performance tests, including the wheel track rutting test, uniaxial penetration test, low-temperature beam bending test, immersion Marshall test, and freeze–thaw splitting test.

### 3.1. Wheel Track Rutting Test

**Figure 8:**
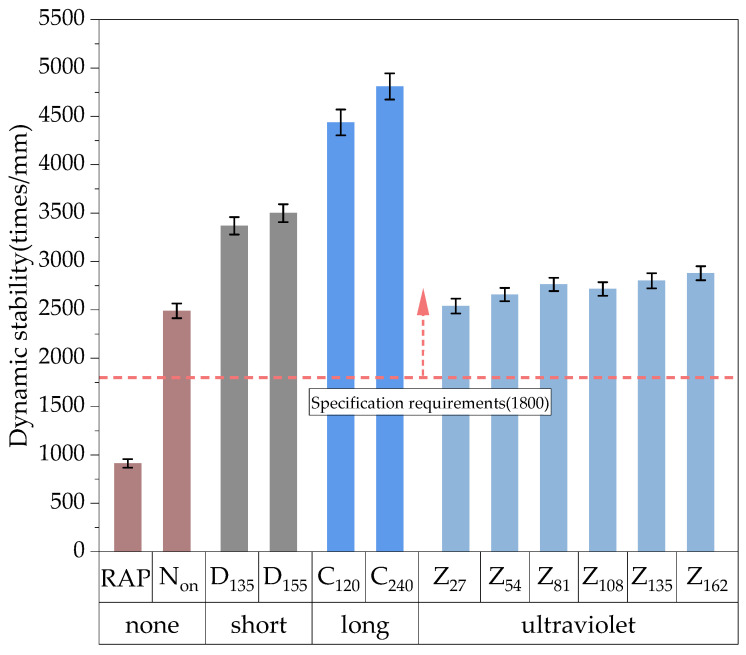
Results of high-temperature dynamic stability.

Following the “Highway Engineering Asphalt and Asphalt Mixture Test Regulations”, wheel track rutting tests were conducted on the 100% RAP and HIR mixture under various aging conditions, using dynamic stability as the evaluation index. Rutting experiments were carried out using a Wheel Track Rutting Instrument from Shengshi Huike Testing Equipment Co., Ltd. (Shanghai, China), a plate specimen sized 300 mm × 300 mm × 50 mm, an experimental temperature of 60 °C, and a wheel pressure of 0.7 MPa. The test results are presented in Figure 8.

**Figure 9:**
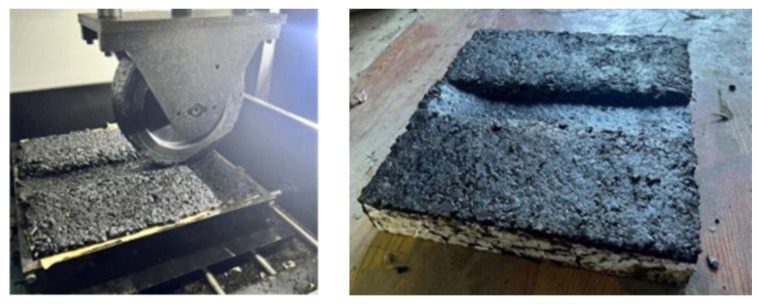
Rutting depth of the original pavement.

**Figure 10:**
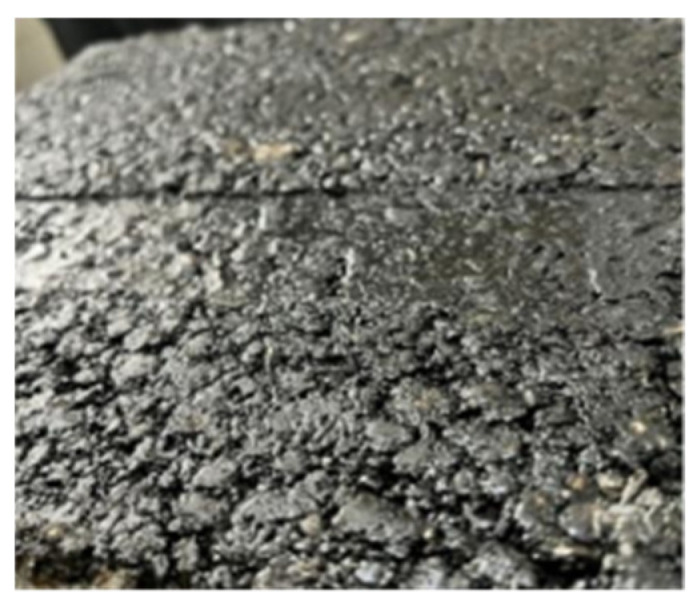
Rutting depth of the composite mixture.

Pavement gradation and asphalt content are critical factors influencing the high-temperature stability of the mixture. The results of the original pavement and the composite mixture after rutting tests are shown in Figure 9 and Figure 10. As shown in Figure 9, the original pavement, with a high asphalt content of 5.45%, exhibits a more significant presence of surface fines and free asphalt, resulting in noticeable binder bleeding and increased mobility. After the rutting test, deeper rutting grooves were observed, likely caused by the excessive asphalt binder content, which reduces the interlocking and occlusion effect between the coarse aggregates. This weakening of the internal skeleton structure makes the mixture more susceptible to deformation under repeated wheel loading. Furthermore, in the fine-graded mixture, a higher asphalt content thickens the asphalt film, coating the mineral material and increasing the mixture’s mobility and temperature sensitivity at high temperatures while reducing its overall stiffness.

As shown in Figure 9, the original pavement exhibited a significant rutting depth, with a dynamic stability (*DS*) of only 913 cycles/mm, indicating poor high-temperature performance. By optimizing the aggregate gradation and controlling the asphalt content, the *DS* increased to 2490 cycles/mm, representing a 172.7% improvement compared to the 100% RAP control group, thus meeting specification requirements. The dynamic stability of the specimen further improved after TO aging. Compared to the non-aged group, the *DS* after short-term aging increased by 35.3%. When the short-term aging temperature reached 155 °C, the *DS* further increased to 3500 cycles/mm. Following long-term aging after short-term aging, the *DS* of the HIR mixture improved significantly. After 240 h of long-term aging, the *DS* reached a maximum of 4809 cycles/mm, a 93.1% increase compared to the non-aged group.

### 3.2. Uniaxial Penetration Test

**Figure 11:**
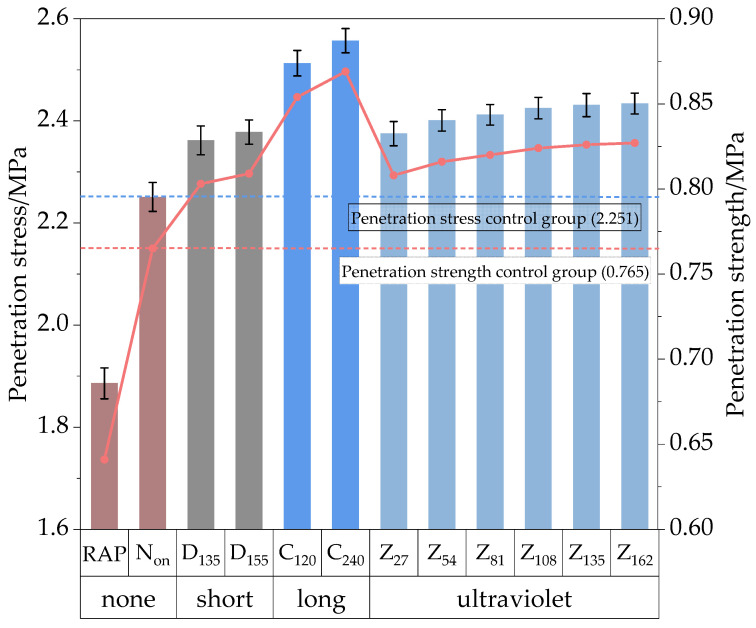
Uniaxial penetration test results.

The uniaxial penetration test was conducted on the HIR mixture using a UTM-25 universal testing machine from Australia IPC Company (Guangzhou, China) to evaluate its high-temperature shear capacity through penetration stress. Marshall specimens were penetrated using a 50 mm long metal rod with a diameter of 28.5 mm, at a penetration rate of 1 mm/min. Specimens should be held in an oven at 60 °C for 6 h to ensure that the test is carried out at the specified temperature. The test results are shown in Figure 11.

As shown in Figure 11, the results of the uniaxial penetration test exhibit a similar increasing trend to those of the rutting test. Both the penetration stress and the penetration strength of the HIR mixture with the optimized gradation were increased by 19.4% compared to the 100% RAP control group. These findings indicate that the high-temperature performance of the HIR mixture was significantly enhanced by incorporating the virgin mixture.

The performance of the HIR mixture was further evaluated under various aging conditions. Compared to the unaged control group, the penetration strength of specimens increased markedly after TO aging. Short-term aging at 135 °C and 155 °C increased penetration strength by 5.0% and 5.8%, respectively, while long-term aging for 120 h and 240 h led to increases of 11.6% and 13.6%, respectively. The high-temperature performance indices of the specimens also improved after UV aging, although the effect was modest. The most notable improvement was observed after 162 h of UV aging, with penetration strength reaching 2.434 MPa.

### 3.3. Low-Temperature Cracking Test

**Figure 12:**
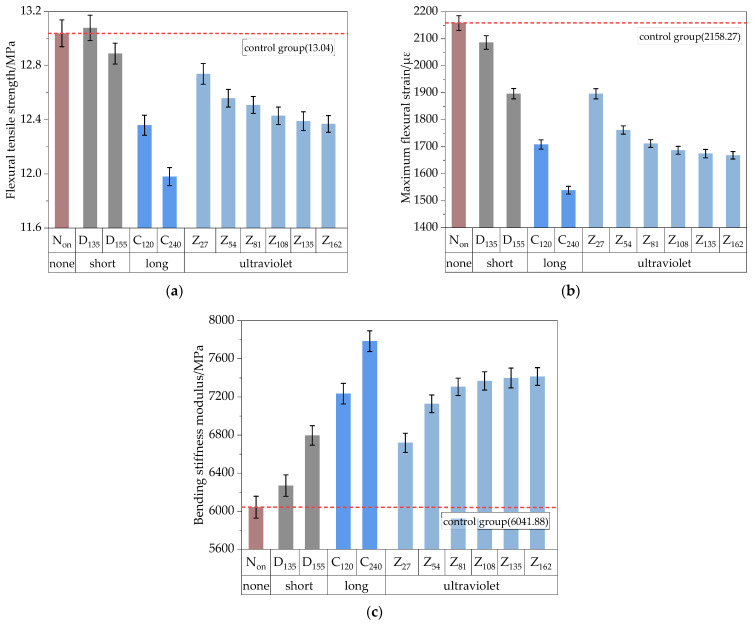
Results of low-temperature beam bending test. (**a**) Flexural tensile strength, (**b**) Maximum flexural strain, (**c**) Bending stiffness modulus.

The trabecular bending test was used to evaluate the low-temperature cracking resistance of the HIR mixture under various aging conditions. The specimens were prepared according to specification requirements. They are rectangular and 100 mm × 10 mm × 10 mm. After being conditioned at −10 °C for 6 h, the specimens were loaded to failure using UTM equipment at a loading rate of 50 mm/min. The results are presented in Figure 12.

As shown in Figure 12, the flexural tensile strength (*R_B_*) and maximum flexural strain (*ε_B_*) of the mixture decreases with increased aging. At the same time, the bending stiffness modulus (*S_B_*) exhibits a corresponding increase. Comparing Group N_on_ to Group D_135_, after short-term aging at 135 °C, the change in *R_B_* was negligible. However, *ε_B_* decreased by 3.4%. The primary distinction between Group D_135_ and Group D_155_ lies in the short-term aging temperature. As the temperature increased, *R_B_* decreased by 1.5%, *ε_B_* dropped to 1896.56 με, and *S_B_* increased by 8.4%. This indicates that elevated temperatures during the construction process negatively impact the low-temperature performance of the mixture, as excessive heating leads to hardening and embrittlement of the HIR mixture, thus reducing its low-temperature performance.

Compared to Group D_135_, Group C_120_ experienced a 5.5% reduction in *R_B_*, and *ε_B_* decreased to 1708.30 με after long-term aging. Comparing Group C_240_ with Group C_120_, after extending the long-term aging duration to 240 h, *R_B_* decreased from 12.36 MPa to 11.98 MPa, while *ε_B_* dropped from 1708.30 με to 1538.98 με. This trend indicates that the low-temperature performance of the HIR mixture declined with prolonged TO aging, while *S_B_* continued to increase.

Comparing Group Z_27_ with Group D_135_, after 27 h of UV aging, the *R_B_* of the HIR mixture decreased by 2.6%, while *ε_B_* declined by 9.1%. The primary distinction among the UV aging control groups lies in the duration of UV exposure. The influence of aging duration on mixture performance becomes evident when comparing Group Z_27_ to Group Z_162_. Specifically, Group Z_54_ exhibited a 1.4% reduction in R_B_ and a 7.1% reduction in *ε_B_* compared to Group Z_27_. As the UV aging duration increased, Group Z_81_ showed a smaller performance decline compared to Group Z_54_, with a 0.4% reduction in *R_B_* and a 3.5% reduction in *ε_B_*. Furthermore, the *R_B_* and *ε_B_* of Group Z_162_ decreased to 12.37 MPa and 1668.23 με, respectively. The results indicate that prolonged UV aging gradually reduced *R_B_* and *ε_B_* in the HIR mixture. However, after 54 h of aging, the rate of change in performance indices slowed, suggesting that the impact of UV aging is most pronounced in the early stages. Meanwhile, the increasing trend in *S_B_* mirrored the changes observed in *R_B_* and *ε_B_*.

### 3.4. Water Stability Performance

The water stability performance of the mixture is typically assessed using the immersion Marshall test and freeze–thaw splitting test. In this work, the water stability performance of the asphalt mixture after TO and UV aging was examined.

(1) Immersing Marshall test

**Figure 13:**
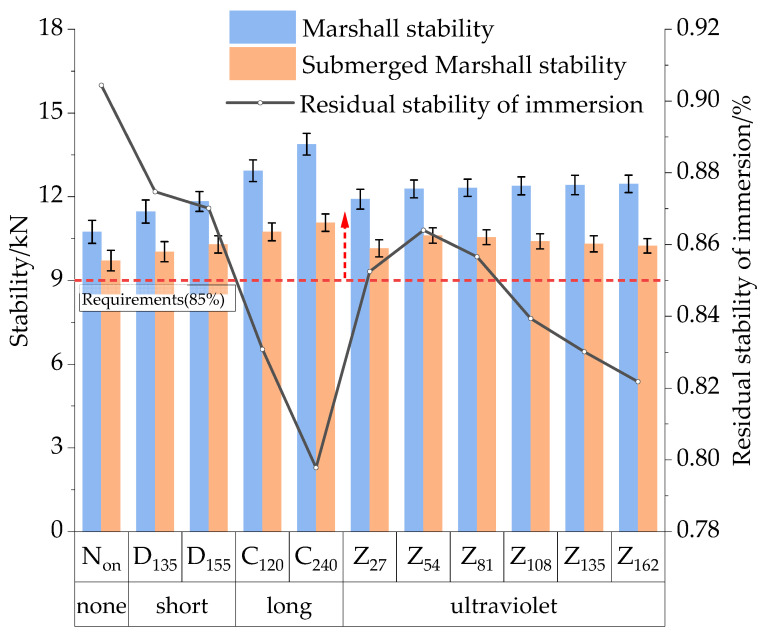
Results of the immersion Marshall test.

The water stability of the HIR mixture under various aging conditions was tested using the immersion Marshall test. To determine its stability, the Marshall specimen was placed in a thermostat tank at 60 °C for 48 h and then compared to the uninsulated specimen. The test results are presented in Figure 13.

The data in Figure 13 indicate that the stability of the HIR mixture increases with aging time and temperature, whereas the residual stability of immersion (*MS*_0_) exhibits a decreasing trend. A comparative analysis of Group Non and Group D_135_ shows that after short-term aging, stability increases by 6.8%. In comparison, *MS_0_* decreases by 3.0%, suggesting that short-term aging reduces the stability of the mixture to some extent. When the short-term aging temperature increases to 155 °C, the mixture stability further improves to 11.83 kN, with a minimal *MS_0_* decrease of only 0.3%. These results suggest that the impact of temperature on the water stability of the asphalt mixture during construction is minimal, causing only a slight reduction in resistance to water damage as the temperature rises.

Further analysis revealed that the stability of Group C_120_ increased from 11.47 kN after short-term aging to 12.93 kN following 120 h of long-term aging, while *MS*_0_ decreased by 4.4%, falling short of the 85% specification. In Group C_240_, where the long-term aging duration was extended to 240 h, stability improved by 7.4%, while *MS*_0_ decreased by 3.3% compared to Group C_120_. This increase in stability can be attributed to the deepening of TO aging, which reduces the lightweight components in the asphalt and enhances the material’s hardness and rigidity. However, the presence of internal defects in the HIR mixture, combined with severe aging, led to compaction difficulties, ultimately contributing to a reduction in *MS*_0_.

A comparison between Group Z_27_ and Group D_135_ reveals that after UV aging, the stability of the specimens increased by 3.8%, while *MS_0_* decreased by 2.2%. When the UV radiation time was extended to 54 h, the specimens exhibited higher stability but lower *MS*_0_. As UV radiation time increased to 81 and 108 h, the performance indices from the immersion Marshall tests stabilized. These results indicate that the performance changes in the mixture are more moderate under UV aging conditions, with the mixture showing higher sensitivity to TO aging in terms of water stability.

(2) Freeze–thaw splitting test

**Figure 14:**
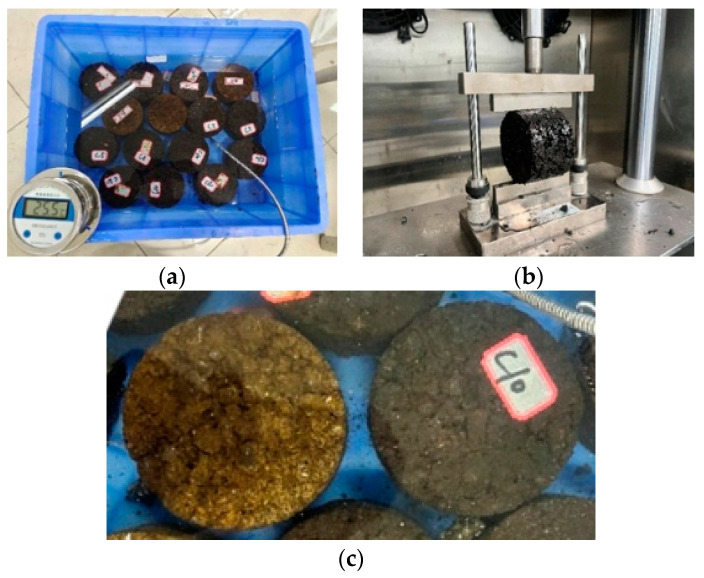
Freeze–thaw splitting setup. (**a**) Specimen insulation, (**b**) Freeze–thaw splitting test, (**c**) Freeze–thaw splitting specimens under UV aging.

The freeze–thaw splitting test was conducted on the HIR mixture under various aging conditions. Marshall specimens were placed in a plastic bag with 10 mL of water and frozen in a refrigerator at −18 °C for 16 h. The specimens were removed and immersed in a thermostat tank at 25 °C for not less than 2 h. Finally, the specimens were loaded with a UTM tester at a rate of 50 mm/min and a temperature of 25 °C. The test procedure is shown in Figure 14.

**Figure 15:**
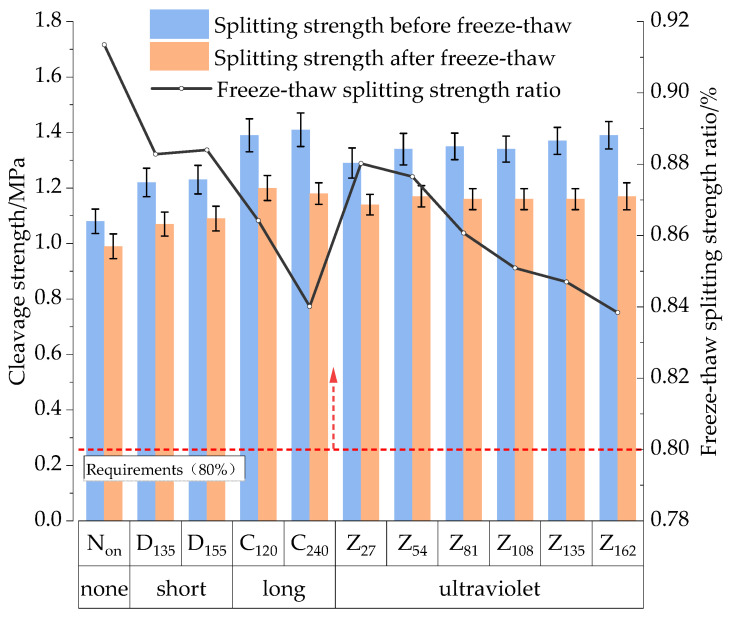
Results of freeze–thaw splitting test.

The water damage resistance was evaluated using the freeze–thaw splitting tensile strength ratio (*TSR*) and the results are shown in Figure 15. As shown in Figure 15, the *TSR* of the aged mixture in the freeze–thaw test meets the specification requirement of not less than 80%. Overall, the splitting strength of the mixture increased, while the *TSR* decreased with progressive aging. Specifically, compared to the Non control group, the pre-freeze–thaw strength of Group D_135_ increased by 12.9%, and the post-freeze–thaw strength of Group D_135_ increased by 8.1%. However, the *TSR* decreased by 4.0%. When the short-term aging temperature rose from 135 °C to 155 °C, performance indices showed no significant changes. In contrast, the long-term aging group C_120_ demonstrated more pronounced changes compared to Group D_135_, with a 13.9% increase in pre-freeze–thaw strength, a 12.2% increase in post-freeze–thaw strength, and a 1.4% decrease in *TSR*, which further declined with extended long-term aging.

Regarding UV aging, Figure 14c shows that the surface of UV-aged specimens turned dark yellow after freeze–thaw cycles, likely due to changes in the chemical structure of the asphalt from prolonged UV radiation, oxidation, and temperature effects. Analyzing the test results, a comparison between Group Z_27_ and Group D_135_ reveals a 5.7% increase in splitting strength after UV aging, while *TSR* slightly decreased. As UV aging time increased, splitting strength gradually improved, but *TSR* continued to decline.

After more than 54 h of UV aging, the splitting strength exhibited no significant variation, while the *TSR* exhibited some fluctuations. This may be attributed to internal defects in the HIR mixture, affecting its homogeneity and performance stability during the UV aging process. Moreover, according to the time–temperature equivalence principle, the material’s behavior over an extended period at low temperatures can be equivalent to its behavior over a shorter period at high temperatures, making it challenging to isolate the influence of the test temperature on long-term UV aging results. The findings indicate that both TO and UV aging significantly impact the water stability of the mixture. As the degree of aging intensifies, the water stability decreases. However, when UV aging reaches a specific duration, its effect on water stability tends to stabilize.

### 3.5. Performance Prediction Model of HIR Mixtures Under Ultraviolet Irradiation

The long-term UV aging test is complex and labor-intensive. In this work, only a one-year UV aging test was simulated. The effects of UV aging over one year remain unclear. Therefore, a model prediction was performed based on the one-year UV aging simulation to estimate the impacts of long-term UV aging. The Verhulst model [30], within the grey system theory, was applied to establish a prediction equation for the HIR mixture pavement performance index under varying UV irradiation durations. Initially, the performance index of the HIR mixture corresponding to different UV irradiation times was selected as the original sequence, as shown in Equation (2). Then, the whitening equation for the Verhulst model can be written as Equation (3). Using the method of separating variables, the general solution of the ordinary differential equation is Equation (4). When *t* = 0, *y*(0) *= y*_0_, let *k = α*/*β*, we can obtain Equation (5). Equation (6) can be calculated by making *ARI_∞_ = k*/*y*_0_. When the time is infinite, the road performance index of the asphalt mixture after UV aging is calculated by Equation (7) [31].

(2) $$y_{i}(t)=[y_{i}(t),y_{i}(t),\cdots ,y_{i}(t)];\;t=1,2,\cdots ,n;\;i=1,2,\cdots ,m$$

(3) $$\frac{dy(t)}{dt}=\alpha y(t)-\beta y^{2}(t)$$

(4) $$y(t)=\frac{\alpha }{\beta (1+Ce^{-\alpha t})}$$

(5) $$y(t)=\frac{k}{1+e^{-\alpha t}(\frac{k}{y_{0}}-1)}$$

(6) $$y(t)=\frac{ARI_{\infty }y_{0}}{1-e^{-\alpha t}(1-ARI_{\infty })}$$

(7) $$\frac{y{\left(t\right)}_{t\to \infty }}{y_{0}}=ARI_{\infty }$$

where *y*(*t*) is the performance evaluation index of the HIR mixture at the time of *t*, the unit of *t* is h; *e* is a transcendental number equal to approximately 2.71828; *ARI_∞_* is the Aging Residual Index; and *α* and *β* are fitting parameters.

As can be seen from the formula, *ARI_∞_* represents the ratio between the pavement performance evaluation index of the asphalt mixture after stabilization by UV aging and its initial value. *ARI_t_* is calculated by Equation (8), which represents the ratio of road performance at a specific aging time to the initial performance and reflects the degree of performance change in the asphalt mixture over that aging period.

(8) $$ARI_{t}=\frac{y(t)}{y_{0}}=\frac{ARI_{\infty }}{1-e^{-\alpha t}(1-ARI_{\infty })}$$

Equation (8) characterizes the relationship between the pavement performance index of the HIR mixture and the time of UV irradiation, and the derivative of Equation (8) can be used to obtain Equation (9), which indicates the rate of change of the pavement performance index of the HIR mixture with the time of UV irradiation.

(9) $$UAR_{t}=\frac{dy(t)}{dt}=\frac{\alpha ARI_{\infty }y_{0}(ARI_{\infty }-1)e^{\alpha t}}{{\left[ARI_{\infty }+e^{\alpha t}-1\right]}^{2}}$$

where *UAR_t_* indicates the rate of change of the pavement performance index of the HIR mixture with the time of UV irradiation.

**Figure 16:**
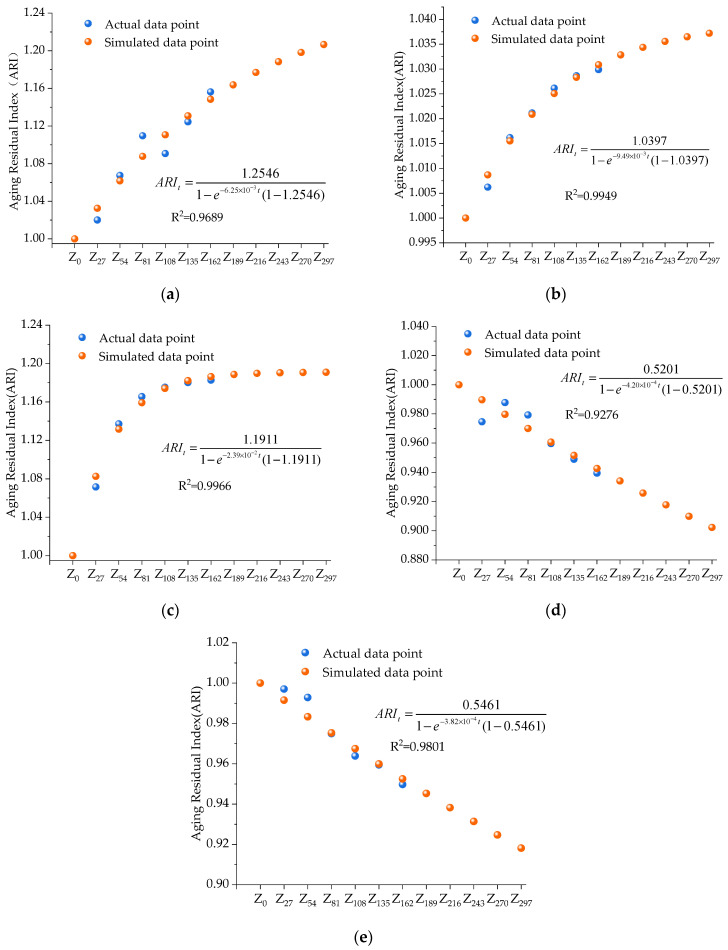
The performance index prediction accuracy of UV aging. (**a**) Dynamic stability, (**b**) Penetration strength, (**c**) Bending stiffness modulus, (**d**) Residual stability of immersion, and (**e**) Freeze–thaw splitting strength ratio.

Equation (8) is applied to predict the performance of the HIR mixture under UV irradiation. The prediction results are presented in Figure 16.

As shown in Figure 16, the UV aging residual index model is well-established, with R-squared values for various pavement performance metrics all exceeding 0.92. Notably, the R-squared values for penetration strength and stiffness modulus even exceed 0.99. The analysis of the aging residual index reveals that dynamic stability, penetration strength, and stiffness modulus demonstrate an increasing trend, with rapid growth in the early stages followed by more moderate growth. As UV irradiation time increases, the aging residual index gradually decreases and eventually levels off. However, the leveling-off period is prolonged, with the final index value remaining relatively low at around 0.5.

**Figure 17:**
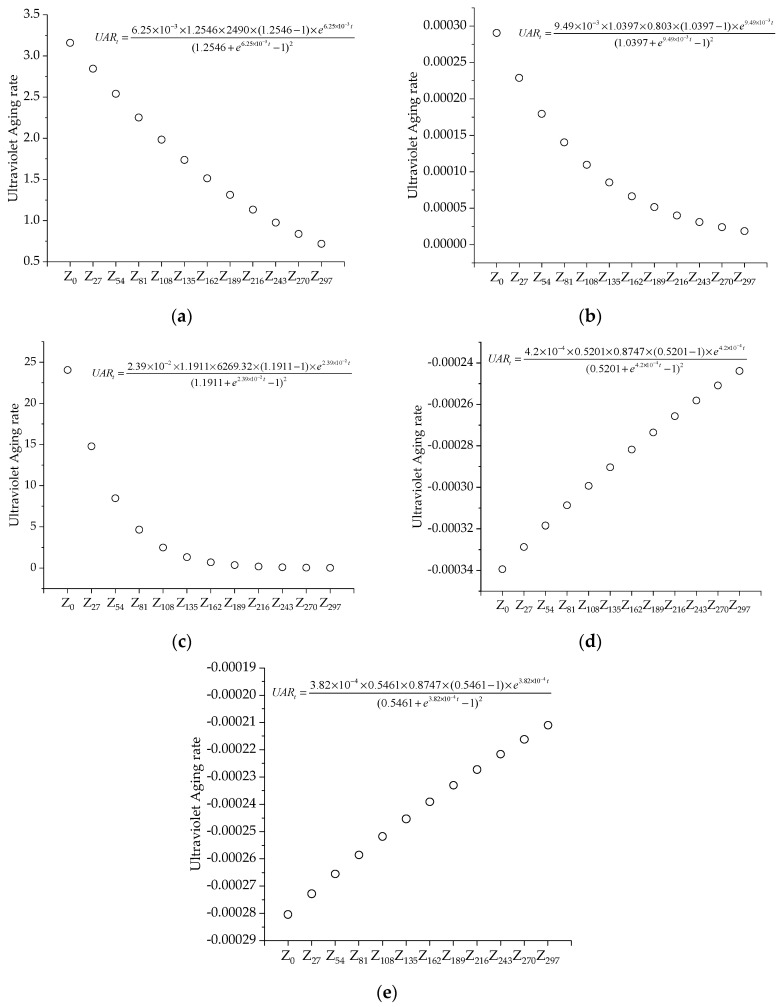
The rate of change of the road performance index with UV irradiation time. (**a**) Dynamic stability, (**b**) Penetration strength, (**c**) Stiffness modulus, (**d**) Residual stability of immersion, and (**e**) Freeze-thaw splitting strength ratio.

As depicted in Figure 17, the UV aging residual index model demonstrates that, apart from water damage resistance, all pavement performance indices experience a rapid initial aging rate, evidenced by a steep curve in the early stages. Specifically, dynamic stability, penetration strength, and stiffness modulus increase sharply at first and then stabilize over time. This is based on the average annual sunshine hours in Yangzhou, Jiangsu Province, China, ranging from 2400 to 3500 h, and calculated using an average indoor UV irradiation intensity of 200 W/m^2^. It is estimated that after approximately 194 h of indoor UV aging, the high- and low-temperature performance indicators of the HIR mixture exhibit minimal changes in the aging rate and residual index. This can be attributed to the presence of reactive double bonds in the asphalt mixtures, which are susceptible to photoreactions. These reactions with UV light result in increased hardness and stiffness, while deteriorating the low-temperature properties. As the irradiation time increases, most of the reactive double bonds on the surface of the asphalt material are consumed, and further changes in material properties slow down. Therefore, in the early stages of aging, strategies should be implemented to reduce the reaction between reactive double bonds and UV light, thus extending the duration of light-induced reactions and mitigating the effects of UV exposure on the road performance of asphalt mixtures.

## 4. Discussion

**Figure 18:**
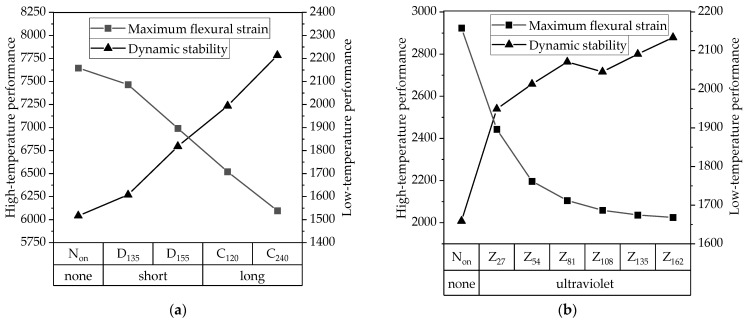
Effect of aging time on high- and low-temperature performance indicators of HIR mixtures. (**a**) TO aging, (**b**) UV aging.

To visually illustrate the effects of aging on high- and low-temperature performance test results, the data from the HIR mixture subjected to different aging methods and durations were plotted as biaxial line graphs, as shown in Figure 18. The figure demonstrates that the high- and low-temperature performance indices of the recycled mixture exhibit opposite trends as aging time increases. Regardless of whether subjected to TO or UV aging, the asphalt mixture shows an increase in high-temperature performance alongside a significant decrease in low-temperature performance. Therefore, greater emphasis should be placed on low-temperature cracking performance during the design process of the HIR mixture while also considering the balance of various properties.

This study primarily focused on recycled asphalt mixtures to examine the effects of aging in commonly used reclaimed materials. Under natural weather conditions, road asphalt materials undergo various effects of aging. The aging effects of TO and UV aging on recycled asphalt were investigated separately. However, the combined aging of TO and UV represents an important area of future research. Combining the effects of UV and TO aging on recycled asphalt mixtures could provide deeper insights into aging mechanisms, thereby improving the aging resistance of asphalt mixtures.

## 5. Conclusions

This study investigated the effects of thermo-oxidative (TO) and ultraviolet (UV) aging on hot in-place recycling (HIR) mixtures through simulations of long-term and short-term oxidative aging and 12 months of UV irradiation. The primary objective was to better understand how these aging processes influence the performance of recycled asphalt pavements, specifically in terms of high-temperature stability, low-temperature crack resistance, and water stability. To achieve this, performance indicators such as dynamic stability, low-temperature flexibility, and resistance to water damage were measured before and after aging, and the Verhulst model was applied to predict performance attenuation under UV aging.

The results showed that both TO and UV aging improved the high-temperature performance of the HIR mixture, with TO aging having a more significant impact. Specifically, 10 years of TO aging resulted in a 78.2% increase in dynamic stability, whereas 12 months of UV aging led to only a 15.6% increase. However, both aging processes led to a decline in low-temperature cracking resistance. Notably, flexural tensile strain decreased by 129.25 με after 10 years of TO aging, while the reduction was less pronounced under UV aging. These findings highlight the trade-off between improved high-temperature performance and reduced low-temperature cracking resistance.

The Verhulst model indicated that UV aging initially significantly impacts pavement performance, underlining the importance of mitigating UV-induced photochemical reactions early in the service life of pavement. Effective aging mitigation strategies are crucial to extending the service life of HIR pavements.

Overall, this study emphasizes the need for a balanced approach in the design of HIR mixtures, considering both high- and low-temperature properties. Future research should focus on improving low-temperature cracking resistance and enhancing aging resistance, particularly in areas with high UV exposure.

## Figures and Tables

**Table 1:** Original pavement gradation.

Percentage Passing Through the Aggregate Sieves/%										
Sieve Size (mm)	16.0	13.2	9.5	4.75	2.36	1.18	0.6	0.3	0.15	0.075
Original gradation	100.0	96.2	77.4	40.3	27.3	21.1	15.8	11.0	8.2	4.9
Upper limit	100.0	100.0	85.0	68.0	50.0	38.0	28.0	20.0	15.0	8.0
Median	100.0	95.0	76.5	53.0	37.0	26.5	19.0	13.5	10.0	6.0
Lower limit	100.0	90.0	68.0	38.0	24.0	15.0	10.0	7.0	5.0	4.0

**Table 2:** Asphalt performance test results.

Technical Indicators	Reclaimed Asphalt Binder	SBS-Modified Asphalt Binder	Requirement	Test Method
Penetration (25 °C,100 g, 5 s)/0.1 mm	42.1	50.1	40~60	T0604
Ductility (5 cm/min, 5 °C)/cm	8.6	29.4	≥20	T0605
Softening point/°C	56.3	77.1	≥60	T0606
Rotational viscosity (135 °C)/Pa·s	2.62	2.75	≤3	T0613

**Table 3:** Basis for classification of reclaimed asphalt aging grades.

Used Material Category	SBS Asphalt Pavement Binder					
Viscosity (Pa·s)	η ≤ 1.6		1.6 < η ≤ 3		η > 3	
Penetration (0.1 mm)	*p* > 30	*p* > 30	20 < *p* ≤ 30	10 < *p* ≤ 20	20 < *p* ≤ 30	10 < *p* ≤ 20
Aging grade	I	II	III	IV	V	VI

**Table 4:** Technical indexes of the RA-102 rejuvenator.

Technical Indicators	RA-102	Requirement	Test Method
90 °C viscosity/cP	4000	/	T0619
Flash point/°C	252	≥220	T0633
Saturation content/%	23.4	≤30	T0618
Aromatic content/%	49	≥30	T0618
The viscosity ratio before and after the RTFOT	1.64	≤3	T0610
Change in mass before and after the RTFOT/%	1.14	≤4%	T0603

**Table 5:** Relative density of coarse aggregates.

Relative Density	Sieve Size/mm			
	13.2~16.0	9.5~13.2	4.75~9.5	2.36~4.75
Apparent relative density	2.732	2.734	2.736	2.735
Relative density of surface drying	2.704	2.705	2.711	2.712
Gross volume relative density	2.691	2.692	2.696	2.699

**Table 6:** Relative density of fine aggregates.

Relative Density	Sieve Size/mm					
	1.18~2.36	0.6~1.18	0.3~0.6	0.15~0.3	0.075~0.1	0~0.075
Apparent relative density	2.749	2.757	2.763	2.771	2.775	2.786

**Table 7:** Technical properties of fillers.

Test Metrics		Test Results	Requirement	Test Method
Water content/%		0.38	≤1	Drying method
Relative density		2.769	≥2.5	T0352
Particle size range	<0.6 mm	100	100	T0351
	<0.15 mm	92.9	90~100	
	<0.075 mm	91.2	75~100	

**Table 8:** Comparison of UV aging test with actual pavement radiation duration.

Lab Aging Time (h)	Simulated Pavement Aging Time (Months)	Lab UV Radiation (MJ/m^2^)
27	2	54.4
54	4	108.8
81	6	163.2
108	8	217.6
135	10	272.0
162	12	326.4

**Table 9:** TO and UV aging test plan.

Group Name	Aging Schemes
N_on_	Blank control group—unaged HIR mixture control group.
D_135_	Short-term aging I—Before molding, the specimen was heated at 135 °C for 4 h.
D_155_	Short-term aging II—Before molding, the specimen was heated at 155 °C for 4 h.
C_120_	Long-term aging I—After short-term aging I, the specimen was heated at 85 °C for 120 h.
C_240_	Long-term aging II—After short-term aging I, the specimen was heated at 85 °C for 240 h.
Z_27_	UV aging I—After short-term aging I, the specimen was UV aged at 50 °C, 556 W/m^2^ radiation for 27 h.
Z_54_	UV aging II—After short-term aging I, the specimen was UV aged at 50 °C, 556 W/m^2^ radiation for 54 h.
Z_81_	UV aging III—After short-term aging I, the specimen was UV aged at 50 °C, 556 W/m^2^ radiation for 81 h.
Z_108_	UV aging IV—After short-term aging I, the specimen was UV aged at 50 °C, 556 W/m^2^ radiation for 108 h.
Z_135_	UV aging V—After short-term aging I, the specimen was UV aged at 50 °C, 556 W/m^2^ radiation for 135 h.
Z_162_	UV aging VI—After short-term aging I, the specimen was UV aged at 50 °C, 556 W/m^2^ radiation for 162 h.

## Data Availability

The original contributions presented in the study are included in the article, further inquiries can be directed to the corresponding author.
